# “Late” Withdrawal Syndrome after Carbamazepine *In Utero* Exposure in a CYP2C9 Slow Metabolizer Newborn

**DOI:** 10.3389/fphar.2017.00217

**Published:** 2017-04-21

**Authors:** Evangelia Passia, Nathalie Rock, Riccardo E. Pfister, Kuntheavy R. Ing Lorenzini, Jules Desmeules, Caroline F. Samer

**Affiliations:** ^1^Department of General Internal Medicine, Geneva University HospitalsGeneva, Switzerland; ^2^Department of Pediatrics, Geneva University HospitalsGeneva, Switzerland; ^3^Clinical Pharmacology and Toxicology, Geneva University Hospitals and Swiss Centre for Applied Human ToxicologyGeneva, Switzerland

**Keywords:** carbamazepine, drug withdrawal, pharmacogenetics, cytochrome P450, epilepsy

## Abstract

We report a case of carbamazepine withdrawal syndrome following *in utero* exposure to carbamazepine related to a pharmacogenetic predisposition factor. The infant was born at 37 1/7 weeks’ gestation by cesarean section to a mother treated for epilepsy with carbamazepine. One hour and thirty minutes after birth, the infant presented a respiratory distress with severe oxygen desaturation requiring intubation 5 h after birth. On the third day of life the infant developed clinical signs of a withdrawal syndrome which resolved progressively after 16 days and symptomatic treatment. The infant genotype analysis showed two low activity CYP2C9 allelic variants (^∗^2/^∗^3 heterozygote) predicting a CYP2C9 slow metabolizer phenotype which could explain reduced carbamazepine elimination and a late and long-lasting withdrawal symptoms observed 3 days after birth. The association of a withdrawal syndrome with carbamazepine exposure has not been previously reported and pharmacogenetic tests might therefore be useful in identifying patients at risk.

## Introduction

Neonatal abstinence syndrome observed in newborns in the neonatal intensive care unit is routinely associated with withdrawal after a prolonged prenatal exposure to opioids. It is, however, possible for children to withdraw from additional medications such as antidepressants or benzodiazepines, or from alcohol. The clinical presentation of withdrawal syndromes varies with the drug, maternal drug history and metabolism, net transfer of drug across the placenta, placental metabolism, and infant metabolism and excretion (Hudak and Tan, 2012).

There is a lack of literature available on withdrawal symptoms in infants of mothers treated with antiepileptics and carbamazepine (CBZ) in particular. CBZ is an iminodibenzyl derivative, structurally similar to tricyclic antidepressants, extensively used in the treatment of epilepsy as well as neuropathic pain and bipolar disorders. It is one of the most commonly used antiepileptic drugs in Europe among women of childbearing age. Recent pharmacoepidemiological studies have shown a prevalence of major malformations of 3.3% after carbamazepine monotherapy in the first trimester with a dose dependent increased risk of spina bifida (0.9% vs. 0.18% in the general population) (Jentink et al., 2010; Tomson et al., 2011; Verrotti et al., 2015) CBZ is furthermore excreted in breast milk.

Acute CBZ toxicity at therapeutic doses mainly affects central nervous and gastrointestinal systems, causing sedation, ataxia, dizziness, nausea, vomiting, constipation and diarrhea. CBZ may also interact with other drugs through the induction of multiple drug metabolizing enzymes.

Carbamazepine is extensively metabolized in the liver by means of cytochromes P450 (CYP) 2C9 and 3A and only 1% of the administered dose is excreted unchanged (Ambrósio et al., 2002). Maternal and infant metabolism of carbamazepine may be affected by genetic polymorphisms modulating CYP activities. CYP2C9 is indeed a polymorphic enzyme and more than 50 allelic variants have been described leading to normal (extensive metabolizers EM) or reduced (poor metabolizers PM) CYP activity^1^. Carriers of at least one CYP2C9 variant may require lower daily doses of various CYP2C9 substrates than wild-type patients (Samer et al., 2013).

This report describes the occurrence of a late and long lasting withdrawal syndrome in a newborn after *in utero* exposure to carbamazepine, possibly related to a pharmacogenetic predisposition factor.

## Patient Description

An infant boy was born at 37 1/7 weeks’ gestation by cesarean section to a G4P1 30 year old mother. The mother had been treated with slow release carbamazepine (Tegretol, Novartis Pharma, Switzerland) for epilepsy since the age of 12, receiving a daily dose of 800 mg/24 h increased to 900 mg/24 h after an epileptic seizure 4 days before delivery. Clonazepam (Rivotril) 0.2 mg was administered once 4 days before delivery. During pregnancy, the mother reported weekly episodes of hypersalivation of 20 minutes duration of probable epileptic origin. She had no other medical comorbid disease and did not receive any other medication.

The infant’s birth weight was 3340 g, head circumference 35 cm, length 52 cm, Apgar score 7/9/10. The adaptation to the extra uterine life was good but required continuous positive airway pressure (CPAP) in room air during 5 min.

One hour and thirty minutes after birth, appearance of groans and desaturation to 50% in room air were observed with nasal flaring and unusual breathing movements. The chest X-ray was compatible with pulmonary immaturity. With symptoms worsening, antibiotherapy (amoxicillin/gentamicin) was initiated, and the infant had to be intubated at 5 h of life and surfactant was administrated at 10 h of life. He was extubated on the 5th day of life and continuous positive airway pressure (CPAP) was used until the 8th day of life. An intermittent support with CPAP was needed until the 20th day of life due to falling saturations and an unusually slow recovery of respiratory distress. Furthermore, from the 3rd day of life, the infant showed major discomfort with irritability, high-pitched cry, yawning and feeding difficulties related to vomiting. Infection, cardiac and digestive disorders were ruled out. Clonazepam administered to the mother 4 days before delivery (0.2 mg single dose) appeared unlikely to have contributed to the withdrawal symptoms. The diagnosis of carbamazepine withdrawal syndrome was then considered as the most likely etiology of the persistent low saturations, feeding difficulties and irritability. The Finnegan scoring system was used to quantify the severity of the withdrawal syndrome symptoms and morphine was administered (day 3 to day 19) in gradually reduced doses according to our usual protocol. No other medications or substances (opioids, alcohol, drugs, and antidepressants) had been administrated to the mother or the infant before these symptoms appeared. The likelihood of adverse drug reaction assessed with the Naranjo algorithm was probable (scoring 6 points).

## Investigations

Investigations were performed to assess for causes of adverse drug reaction to carbamazepine. They included CYP2C9 genotyping and CYP phenotyping assessments.

### CYP2C9 Genotyping

Genomic DNA was extracted from whole blood (200 μl) using the QIAamp DNA blood mini kit (QIAGEN, Hombrechtikon, Switzerland). CYP2C9^∗^2 (rs1799853, g.3608C > T) and CYP2C9^∗^3 (rs1057910, g.42614A > C) were determined in a single multiplex PCR, with fluorescent probe melting temperature analysis on a LightCycler (Roche, Rotkreuz, Switzerland) as previously described Burian et al. (2002).

### CYP Phenotyping

The activity of six CYP and *P*-glycoprotein (P-gp) was simultaneously assessed on a dried blood spot using microdoses of specific probe drugs of CYP1A2, 2B6, 2C9, 2C19, 2D6, 3A4/5 and P-gp (caffeine, bupropion, flurbiprofen, omeprazole, dextromethorphan, midazolam and fexofenadine respectivily) as previously described by Bosilkovska et al. (2014).

## Results

Pharmacogenetic testing of CYP2C9, the polymorphic enzyme responsible for the metabolism of carbamazepine, showed that the infant was a carrier of two reduced activity allelic variants (^∗^2/^∗^3 heterozygote) of CYP2C9 (g.3608CT, g.42614AC) which predicts a status of slow metabolizer of CYP2C9.

The mother was also carrying two allelic variants (^∗^2/^∗^2 homozygote) of CYP2C9 (g.3608TT) but CYP2C9 induction by carbamazepine counteracted this genetic defect. Hence, resulting CYP2C9 activity was normal and plasma carbamazepine levels were thus in the therapeutic range during pregnancy (value 30 μmol/L and 24 μmol/L, therapeutic range: 17–42 μmol/L) for a daily dose of 800 mg/24 h. CYP3A4 activity was normal.

## Discussion

This is the first published report of an infant developing a late and long lasting withdrawal syndrome following *in utero* carbamazepine exposure.

A withdrawal syndrome was recently reported with oxcarbazepine, a structural derivative of CBZ, in an infant born at 35 weeks’ gestation by urgent cesarean section to a mother in status epilepticus who had been treated with oxcarbazepine throughout her pregnancy (Rolnitsky et al., 2013). On the third day of life the infant developed clinical signs of a withdrawal syndrome, which peaked at day 7 and resolved by day 12. Follow-up showed normal development at 15 months. A clinical study of 57 cases of fetal anticonvulsant syndromes (CBZ was used in monotherapy in 4 cases) has reported 11 cases of withdrawal syndromes but data on specific molecules involved were missing (Moore et al., 2000). Recently, a case of neonatal gabapentine withdrawal syndrome has also been reported (Carrasco et al., 2015).

Carbamazepine is extensively metabolized in the liver by means of cytochromes P450 (CYP) 3A and CYP2C9. Genetic polymorphisms modulating drug-metabolizing enzyme activities such as CYP explain part of the variability in drug responses (Samer et al., 2013). CYP2C9 is a polymorphic enzyme and more than 50 allelic variants have been described^2^. On the basis of CYP2C9 activity, extensive (EM) and poor (PM) metabolizers can be distinguished. The two most common allelic variants associated with a reduced activity of CYP2C9 and a phenotype of slow metabolizer are CYP2C9^∗^2 (rs1799853, g.3608C > T) and CYP2C9^∗^3 (rs1057910, g.42614A > C). These two alleles are carried by approximately 35% of Caucasians (Samer et al., 2013). Carriers of at least one CYP2C9^∗^2 or CYP2C9^∗^3 allele require lower daily doses of CYP2C9 substrates such as vitamin K antagonists and are prone to adverse drug reactions with some non-steroidal anti-inflammatory drugs (Samer et al., 2013).

Our patient was a carrier of two allelic variants (^∗^2/^∗^3 heterozygote) of CYP2C9, which predicts a phenotype of a slow metabolizer of CYP2C9. The reduced activity of CYP2C9 may have led to reduced elimination of carbamazepine and higher plasma levels despite standard maternal doses, which may consequently be responsible for the unusual and delayed withdrawal symptoms 3 days after birth.

CYP2C9 genotyping as well as CYP450 phenotyping using the Geneva cocktail were assessed in the mother (Bosilkovska et al., 2014). She was carrying two CYP2C9 variant alleles (^∗^2/^∗^2 homozygote), compatible with a poor metabolizer genotype for CYP2C9. However, phenotypic CYP2C9 activity was normal as CYP2C9 induction by carbamazepine counteracted the genetic defect. CYP phenotyping indeed offers the advantage to provide information on the real-time (*in vivo*) activity of CYP enzymes and may therefore provide the most clinically relevant information as it reflects a combination of genetic, environmental and endogenous factors (Samer et al., 2013).

Immaturity of some drug-metabolizing enzymes at birth may be responsible for reduced clearance and increased half-life, and may have contributed to the clinical manifestations described in the case of our newborn. However, ontogeny of liver metabolism would be relevant to all newborns from carbamazepine treated mothers and carbamazepine has not been previously associated with withdrawal syndromes after exposure during pregnancy. We therefore believe that the role of the pharmacogenetic factor was predominant in this situation.

## Conclusion

We describe the case of a newborn to a carbamazepine-treated mother who developed symptoms of late and long lasting withdrawal syndrome. The genotype analysis shows that the infant is a poor metabolizer of CYP2C9, responsible for a slower metabolism of carbamazepine and predisposition to withdrawal symptoms. The association of a neonatal withdrawal syndrome with carbamazepine exposure has not been previously reported and should lead to consideration of pharmacogenetic tests to identify patients at risk for a causal explanation and planning of future treatments.

## Ethics Statement

Our submission is a case report. Data was collected during the routine clinical care of the patient and her mother. In accordance with the Declaration of Helsinki, written informed consent was provided by the mother for herself and for her child, and confidentiality of the data has been ensured. Ethics approval was not required by our ethical board in this situation.

## Author Contributions

EP was in charge of the pharmacological investigations done in the patient and wrote the manuscript. NR was involved in the care of the patient and wrote the manuscript. RP was involved in the care of the patient and wrote the manuscript. KI was involved in the interpretation of the results and wrote the manuscript. JD supervised the investigations and the redaction of the manuscript. CS interpreted the results and wrote the manuscript. All authors read and approved the manuscript.

## Conflict of Interest Statement

The authors declare that the research was conducted in the absence of any commercial or financial relationships that could be construed as a potential conflict of interest.

## Footnotes

1 http://www.cypalleles.ki.se/cyp2c9.htm

2 http://www.cypalleles.ki.se/cyp2c9.htm
